# Effect of Zebularine on p16INK4a, p14ARF, p15INK4b, and DNA Methyltransferase 1 Gene Expression, Cell Growth Inhibition, and Apoptosis Induction in Human Hepatocellular Carcinoma PLC/PRF5 and Pancreatic Cancer PA-TU-8902 Cell Lines

**DOI:** 10.22037/ijpr.2020.112223.13614

**Published:** 2020

**Authors:** Masumeh Sanaei, Fraidoon Kavoosi, Farzane Hosseini

**Affiliations:** a *Research Center for Non-Communicable Diseases, Jahrom University of Medical Sciences, Jahrom, Iran. *; b *Student of Research Committee, Jahrom University of Medical Sciences, Jahrom, Iran.*

**Keywords:** INK4 CDKI, DNA-methyltransferase 1, Zebularine, Cancer

## Abstract

Tumorigenesis must be understood as a summary of altered genetic and genomic changes resulting in the inactivation of tumor suppressor genes (TSGs). One of the characterizations of epigenetic alterations is DNA methylation. Epigenetic alteration of the p16INK4a, p14ARF, p15INK4b*, *and DNA methyltransferase 1 gene (DNMT1) expression occurs in hepatocellular carcinoma (HCC) and pancreatic cancer frequently. DNA methyltransferase inhibitors (DNMTIs), such as zebularine, play a significant effect on the demethylation and reactivation of TSGs. This study aimed to investigate the effect of zebularine on p16INK4a, p14ARF, p15INK4b, and DNA methyltransferase 1 gene expression, cell growth inhibition, and apoptosis induction in HCC PLC/PRF5 and pancreatic cancer PA-TU-8902 cell lines. Both cell lines were cultured and treated with zebularine at different times. The MTT assay, real-time quantitative reverse-transcription polymerase chain reaction (qRT-PCR), and flow cytometry were used to determine cell viability, gene expression, and apoptotic cells, respectively. The result indicated that zebularine inhibited cell growth of both cell lines significantly as time- and dose-dependent manner (*P < *0.007). The agent induced significant down-regulation of DNMT1 and up-regulation of p16INK4a, p14ARF, p15INK4b (*P < *0.028). Besides, it had a significant apoptosis effect on both cell lines (*P < *0.001). This compound had a strong significant effect on PLC/PRF5 in comparison to PA-TU-8902 cells. Concluding, zebularine inhibited PLC/PRF5 and PA-TU-8902 cell growth and induced apoptosis in these cell lines. The most likely mechanism underlying the zebularine played its role involves down-regulation of DNMT1 and up-regulation of p16INK4a, p14ARF, and p15INK4b genes.

## Introduction

Hepatocellular carcinoma (HCC) and pancreatic cancers are lethal human cancers with a wide geographical variation (1, 2). As for many other cancers, the development of these cancers must be understood as a summary of altered genetic and genomic changes and also epigenetic alterations in cell cycle regulatory genes resulting in activation of oncogenes and inactivation of tumor suppressor genes (TSGs). In contrast to genetic events, epigenetic alteration is a reversible change characterized by three mechanisms, including DNA hypermethylation, DNA hypomethylation, and histone modifications affecting chromatin conformation without any change in the structure of DNA sequences (3). Epigenetic alterations of the INK4alpha/ARF or CDKN2A-locus could occur in HCC. The INK4a-ARF locus, located on 9p21, codes three important TSGs, p14ARF, p15INK4b, and p16INK4a, involved in cell-cycle regulation. One of the characterizations of genomic cancer is the DNA methylation change that has also been termed epigenetic alterations. Epigenetic alterations of p14ARF, p15INK4b, and p16INK4a have been demonstrated in HCC and pancreatic cancer (4-6). In addition to HCC and pancreatic cancers, hypermethylation of these genes has been reported in other cancers such as pulmonary squamous cell carcinoma (SqCC), cervical cancer, prostate cancer, and esophageal carcinoma (7-10). DNA hypermethylation is reversible by DNA demethylating agents. Several classes of chemical agents, including adenosine analogs, nucleotide analogs, aminobenzoic derivatives, hydrazines, polyphenols, disulfides, phthalides, and antisenses, are evaluated as DNMTIs targeting DNA hypermethylation. These compounds can induce demethylation, which leads to reactivation of hypermethylated TSGs resulting in apoptosis induction in cancer cells. The significant effect of DNA methyltransferase inhibitors (DNMTIs), such as 5-aza-2′-deoxycytidine (5-aza-CdR) and zebularine [1-(beta--ribofuranosyl)-1, 2-dihydropyrimidin-2-one] on HCC (11, 13), and pancreatic cancers (14-16) has been reported by several studies. Consequently, DNA demethylation is the molecular mechanism by which the demethylating agents such as zebularine and 5-aza-CdR affect silenced TSGs. Indeed, zebularine can restore silenced TSGs by inhibition of DNA methyltransferase 1 (DNMT1) activity. An experimental study has demonstrated that this agent incorporates into DNA and exhibits cell growth inhibition by DNMT1 inhibition in human cancer cell lines, including T24 bladder cancer, SW48, HCT15, and HT-29 colon cancer, PC3 prostate cancer, CFPAC-1 pancreatic cancer, and CALU-1 lung cancer (17). A preclinical study has indicated that mRNA expression of p14ARF could be reactivated in methylated neuroblastoma cells (18). Other researches have reported that zebularine is an effective inhibitor of p15INK4B and p16INK4a methylation and reactivates these methylated genes leads to cell growth inhibition in AML and T24 bladder carcinoma cells, respectively (19, 20). In addition to zebularine, it has been shown that DNA demethylating agent 5-aza-CdR can reactivate methylated p16INK4a in HCC HepG2 ‘and HuH6, HuH7, and HLF cell lines (21, 22). Previously, we reported the effect of 5-aza-CdR on DNMT1 gene expression and apoptosis induction in the HCC WCH-17 cell line (23). The results of other researchers and our previous results encouraged us to design the present study. The aim of this study was to investigate the effect of zebularine on p16INK4a, p14ARF, p15INK4b, and DNA methyltransferase 1 gene expression, cell growth inhibition, and apoptosis induction in human hepatocellular carcinoma PLC/PRF5 and pancreatic cancer PA-TU-8902 cell lines.

## Experimental

*Materials*

Hepatocellular carcinoma PLC/PRF5 and pancreatic cancer PA-TU-8902 cell lines were purchased from the National Cell Bank of Iran-Pasteur Institute and cultured and maintained in Dulbecco’s modified Eagle’s medium (DMEM) supplemented with fetal bovine serum 10% and antibiotics in a humidified atmosphere of 5% CO2 in air at 37 ◦C. Zebularin was provided from Sigma (St. Louis, MO, USA) and dissolved in distilled water as a stock solution. All other experimental solutions were obtained by diluting the provided stock solution. Other agents including, DMSO, antibiotics, 3-[4, 5-dimethyl-2-thiazolyl]-2, 5-diphenyl-2H-tetrazolium bromide (MTT), trypsin-EDTA, Phosphate-buffered saline (PBS), Annexin-V-(FITC), propidium iodide (PI), DMEM were purchased from Sigma. Total RNA extraction kit (TRIZOL reagent) and real-time polymerase chain reaction (PCR) kits (qPCR MasterMix Plus for SYBR Green I dNTP) were obtained from Applied Biosystems Inc. (Foster, CA, USA). This work was approved by the Ethics Committee of Jahrom University of Medical Science with a code number of IR.JUMS.REC.1398.022. 

*Cell culture and cell viability*

The effect of zebularine on PLC/PRF5 and PA-TU-8902 cell viability was investigated using MTT assay, based on the previously described method (23). Briefly, 5 × 10^5 ^cells per well were treated with various concentrations of zebularine (0, 10, 25, 50, 75, 100, 250, and 500 μM). After 24 and 48 h of incubation, MTT (0.5 mg/mL PBS) was added to each well and incubated at 37 °C for 3 h to determine the number of living cells. Then the formed formazan crystals were dissolved in DMSO and shaken for 10 min to dissolve all of the crystals. Finally, the optical density was detected by a microplate reader at a wavelength of 570 nM. Each experiment was repeated three times (triplicates).

*Cell apoptosis assay*

An apoptosis assay was performed to obtain the apoptotic effect of zebularine on PLC/PRF5 and PA-TU-8902 cells, based on the previously described method (24). Briefly, 5 × 10^5^ in 24-well plates were seeded in triplicate and incubated overnight. Then, the PLC/PRF5 and PA-TU-8902 cells were treated with zebularine in indicated concentration (74.65 and 98.82 μM respectively), according to IC_50 _values, for 24 and 48 h, the control groups were incubated with medium + distilled water, distilled water equivalent to the drug solvent was used. Following the staining of the samples using annexin V-FITC and PI, they were analyzed by FACScan flow cytometry (Becton Dickinson, Heidelberg, Germany).

*Real-time quantitative reverse-transcription polymerase chain reaction*

*(qRT-PCR) analysis*

To determine whether zebularine could affect p16INK4a, p14ARF, p15INK4b, and DNA methyltransferase 1 gene expression, qRT-PCR was performed. After treatment times (24 and 48 h), total RNA was isolated from PLC/PRF5 and PA-TU-8902 cells treated with zebularine (74.65 and 98.82 μM respectively) using Trizol reagent (Invitrogen), and cDNA was synthesized from total RNA with Superscript III reverse transcriptase (Invitrogen). The expression of mRNAs was measured by quantitative real-time PCR using StepOnePlus (Applied Biosystem, USA) instrument and SYBER green PCR kit (TaKaRa Bio). The thermocycling condition and the amplification reactions were performed as mentioned previously (26). The primer sequences of the genes are shown in Table 1. GAPDH was used as an endogenous control. Data were analyzed using the comparative Ct (ΔΔct) method.

*Statistical analysis*

The database was set up with the SPSS 16.0 software package (SPSS Inc., Chicago, Illinois, USA) for analysis. The data were acquired from three tests and are shown as means ± standard deviations. Statistical comparisons between groups were performed with ANOVA (oneway ANOVA) and Turkey test. A significant difference was considered as *P < *0.05.

## Results

*Cell viability*

**Figure 1:**
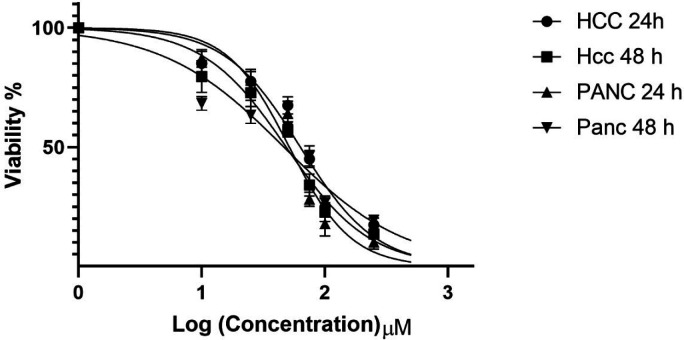
In-vitro effects of zebularine (0, 10, 25, 50, 75, 100, 250, and 500 μM) on PLC/PRF5 and PA-TU-8902 cell viability evaluated by MTT Assay at different times (24 and 48 h). Values are means of three experiments in triplicate. The zebularine had a dose- and time-dependent effect, statistical comparisons between groups were performed with ANOVA (One‑way ANOVA) and Turkey test

The viability of Hepatocellular carcinoma PLC/PRF5 and pancreatic cancer PA-TU-8902 cell lines treated with zebularine (10, 25, 50, 75, 100, 250, and 500 μM) was determined by MTT assay as mentioned in the method section. The inhibitory effect of zebularine on both PLC/PRF5 and PA-TU-8902 cell lines was dependent on the dose and incubation time, as shown in Figure 1. This agent demonstrated a significant inhibitory effect with all used doses (*P* < 0.003). IC_50_ values were obtained with approximately 74.65 and 98.82 μM, as a mean of 24 and 48 h, for PLC/PRF5 and PA-TU-8902 cell lines, respectively. 

*Cell apoptosis*

**Figure 2:**
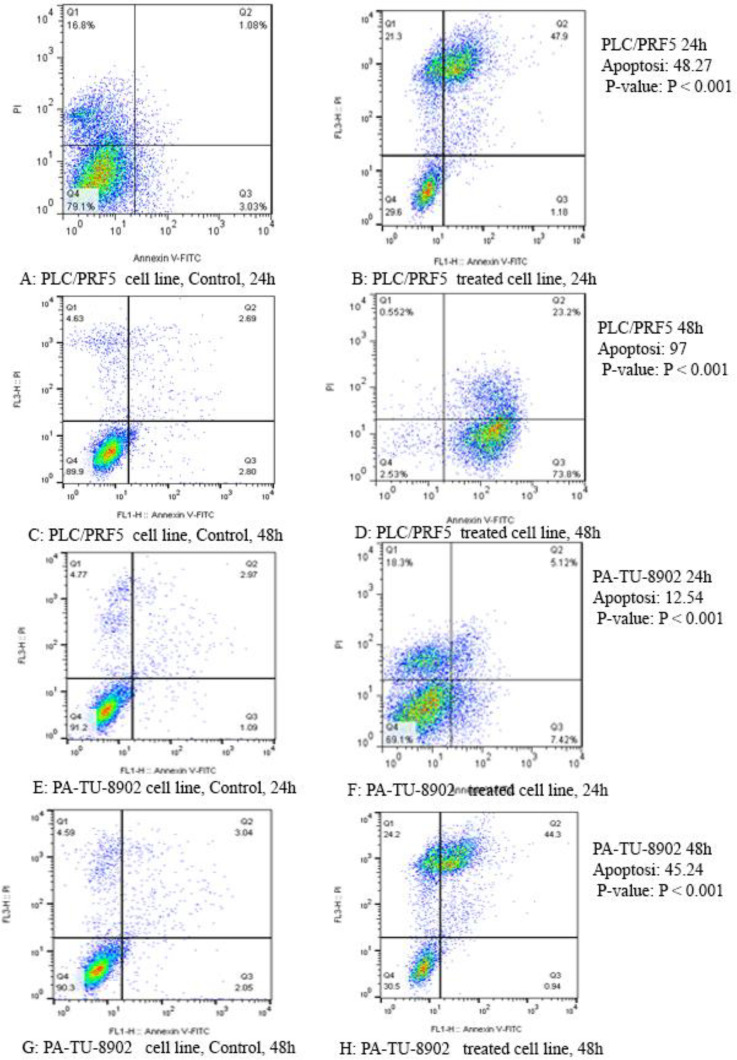
The apoptotic effect of zebularine on PLC/PRF5 and PA-TU-8902 cells (74.65 and 98.82 μM respectively) versus control groups at 24 and 48 h. The cells were treated with zebularine for 24 and 48 h, and the apoptotic effect was investigated by flow cytometric analysis. Results were obtained from three independent experiments and were expressed as mean ± standard error of the mean. The upper right quadrant of each figure shows the percentage of cells in late apoptosis, the lower right quadrant shows the percentage of cells in early apoptosis, the upper left quadrant shows the percentage of necrotic cells, and the lower left quadrant shows the percentage of viable cells. The zebularine induced apoptosis of both cell lines significantly in a time-dependent manner (*P *< 0.001)

**Figure 3:**
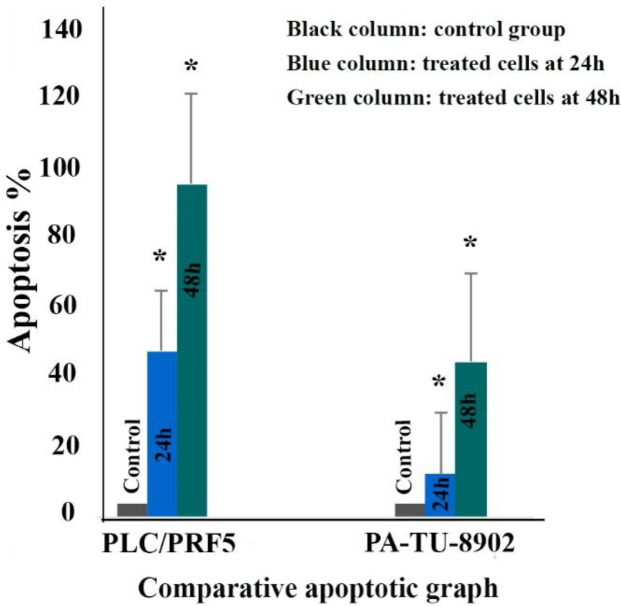
The comparative effects of zebularine at a concentration of 74.65 μM on PLC/PRF5 cells compared to PA-TU-8902 cells treated with zebularine at a concentration of 98.82 μM. The first column of each group belongs to the control group and the others belong to treated cells with the zebularine with the mentioned concentrations at 24 and 48 h. Asterisks (*) indicate significant differences between the treated and untreated control groups. As shown above, zebularine indicated a more significant apoptotic effect on PLC/PRF5 cells in comparison to PA-TU-8902 cells

To determine whether zebularine could induce apoptosis, the PLC/PRF5 and PA-TU-8902 cells were stained using annexin-V-(FITC), as mentioned in the method section. As depicted in Figure 2, significant differences were observed by comparing the amounts of annexin V-single positive zebularine-treated cells to the untreated control groups in both cell lines. The apoptotic effect of the agent on PLC/PRF5 cells in comparison to PA-TU-8902 cells was more significant. Besides, zebularine induced apoptosis in both cell lines in a time-dependent manner, Table 2 and Figure 3. In the apoptotic graph, the upper right quadrant shows the percentage of cells in late apoptosis, the lower right quadrant shows the percentage of cells in early apoptosis, the upper left quadrant shows the percentage of necrotic cells, and the lower left quadrant shows the percentage of viable cells.

*Gene expression*

**Figure 4:**
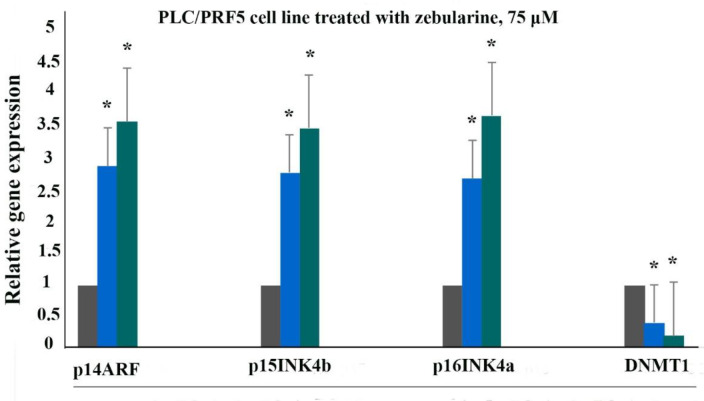
The relative expression level of p16INK4a, p14ARF, p15INK4b, and DNMT1 genes in the PLC/PRF5 cells treated with zebularine (74.65 μM) versus control groups at 24 and 48 h. The first column of each group belongs to the control group and the others belong to the treated cells with zebularine at 24 and 48 h. Asterisks (*) indicate significant differences between the treated and untreated groups

**Figure 5:**
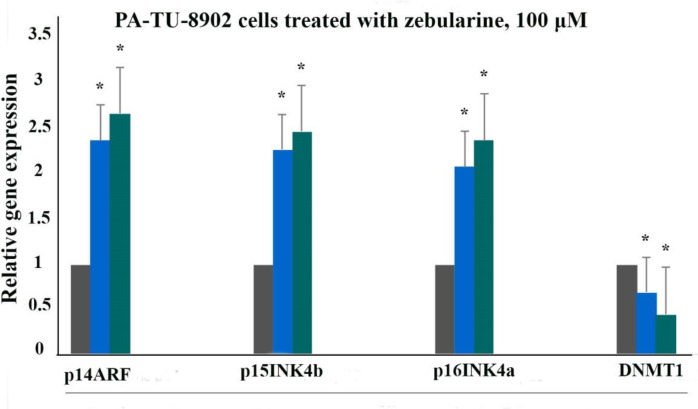
The relative expression level of p16INK4a, p14ARF, p15INK4b, and DNMT1 genes in the PA-TU-8902 cells treated with zebularine (98.82 μM) versus control groups at 24 and 48 h. The first column of each group belongs to the control group and the others belong to treated cells with the zebularine at 24 and 48 h. Asterisks (*) indicate significant differences between the treated and untreated groups

 The effect of zebularine on p16INK4a, p14ARF, p15INK4b, and DNA methyltransferase 1 gene expression was evaluated by quantitative real-time RT-PCR analysis. The result indicated that treatment with zebularine (24 and 48 h) upregulated p16INK4a, p14ARF, p15INK4b, and down-regulate DNMT1 gene expression significantly in both cell lines, the PLC/PRF5 and PA-TU-8902, Table 3, Figures 4 and 5. The effect of zebularine on the gene expression in PLC/PRF5 cells in comparison to PA-TU-8902 cells was more significant.

## Discussion

Cell cycle progression and regulation is a highly-regulated process that involves multiple mechanisms and checkpoints. The molecular mechanism of various stages of the cell cycle has been evaluated during the past decade. The CDKs are a family of enzymes that form the heart of the regulatory machine during the cell cycle progression. The active form of these enzymes includes a complex of two proteins, a kinase, and a cyclin, which form positive regulators and induce cell cycle progression. Whereas CKIs account for the important negative regulators which stop cell cycle progression in response to multiple regulatory signals. The deregulation of the cell cycle regulatory genes such as CDKs is one of the most frequent alterations during tumorigenesis and cancer development (31). The hypermethylation of CDKIs such as the CIP/KIP family has been shown in several cancers (32, 33). Indeed, the silencing of the CDKIs promoter by hypermethylation plays an important role in cancer induction and tumor development. *In-vitro* studies have indicated that DNA demethylating agents can reactivate silenced TSGs such as p14^ARF^*, *p16^INK4A^, and p53 (34, 35). In the present study, zebularine inhibited cell growth and induced apoptosis in both PLC/PRF5 and PA-TU-8902 cell lines. Subsequently, we evaluated the molecular mechanism of this effect and found that it upregulated p16INK4a, p14ARF, and p15INK4b and down-regulated DNA methyltransferase 1 gene expression significantly. Additionally, our work demonstrated that zebularine had a more significant effect on PLC/PRF5 in comparison to TU-8902 cells. A similar pathway has been reported for zebularine in other cancers. It has been reported that this compound upregulates p16, p21, and p27gene expression by inhibition of DNMT1 activity in bladder transitional carcinoma cells T24, pancreatic carcinoma CFPAC-1 cells, colon carcinoma HCT15, SW48, and HT-29, and lung carcinoma CALU-1 cell lines (17). Another study has been shown that zebularine down-regulates CDK2 and upregulates p21WAF/CIP1 and p53 in HCC HepG2 (12). In acute myeloid leukemia (AML), AML193, zebularine treatment results in a dose-dependent increase in p15INK4B expression and apoptosis induction (19). Increased expression of the silenced cell cycle regulatory genes after inhibition of DNMTs by DNA demethylating agent 5-aza-CdR has been reported by several studies. By this pathway, DNMTs inhibition, zebularine increases p53/p21Waf1/Cip1 expression in A549 cells (wild-type p53) (36). In esophageal cancer cell lines, this compound can increase p27kip1 mRNA expression through DNA demethylation (37). An important molecular mechanism of INK4 family, including p16INK4a, p15INK4b, p18INK4c, and p19INK4d, is the cyclin D-Cdk4-6/INK4/Rb/E2F pathway, which plays a significant role in controlling cell growth by integrating multiple antimitogenic and mitogenic stimuli. This family, INK4 family, blocks the cell cycle progression by binding to either Cdk4 or Cdk6 and inhibiting the action of cyclin D (38). In addition to the INK4 family, DNMTIs can induce apoptosis by reactivation of Cip/Kip family, including p21Cip1, p27Kip1, and p57Kip2 (39). *In-vitro* study has been shown that DNA methyltransferase inhibitor 5-aza-CdR can induce G2/M cell cycle arrest in leukemia cells by p21WAF1/CIP1 up-regulation (40). Overexpression of p57 has been reported in pancreatic cancer cell lines after treatment with 5-aza-CdR (41). Besides, this compound can restore the P21 gene in HCC, a major mechanism causing cell cycle arrest in this cell line (42).

In summary, DNA methyltransferase inhibitors can inhibit cell growth and induce apoptosis by reactivation of cell cycle regulatory genes, INK4, and the Cip/Kip family. We did not evaluate the effect of 5-aza-CdR on the Cip/Kip family in HCC and pancreatic cancer. Therefore, this evaluation is recommended.

**Table 1:** The primer sequences of p16INK4a, p14ARF, p15INK4b, and DNMT1 genes

**Primer**	**Primer sequences (5' to 3')**	**Reference**
p14^ARF^ Forward Reverse	GTGGGTTTTAGTTTGTAGTT AAACCTTTCCTACCTAATCT	
p15INK4b Forward Reverse	AAGCTGAGCCCAGGT CTCCTA CCACCGTTGGCCGTAAACT	
p16INK4a Forward Reverse	CCCGCTTTCGTAGTTTTCAT TTATTTGAGCTTTGGTTCTG	
DNMT1 Forward Reverse	GAG GAA GCT GCT AAG GAC TAG TTC ACT CCA CAA TTT GAT CAC TAA ATC	

**Table 2:** The percentage of apoptotic cells treated with zebularine at different periods. A significant difference was considered as *P *< 0.05. *P*-value: *P *< 0.001.

**Cell line**	**Drug**	**Dose (μM )**	**Duration (h)**	**Apoptosis (%)**	***P*** **-value**
PLC/PRF5	zebularine	74.65	24	48.27	0.001
PLC/PRF5	zebularine	74.65	48	97	0.001
PA-TU-8902	zebularine	98.82	24	12.54	0.001
PA-TU-8902	zebularine	98.82	48	45.24	0.001

**Table 3:** The relative expression level of p16INK4a, p14ARF, p15INK4b, and DNMT1 genes in treated cell groups in comparison to untreated control groups. A significant difference was considered as *P *< 0.05

**Cell line**	**Gene**	**Drug**	**Dose (μM)**	**Duration (h)**	**Expression**	***P*** **-value**
PLC/PRF5	p14ARF	zebularine	74.65 μM	24	2.9	0.001
PLC/PRF5	p14ARF	zebularine	74.65 μM	48	3.6	0.001
PLC/PRF5	p15INK4b	zebularine	74.65 μM	24	2.8	0.001
PLC/PRF5	p15INK4b	zebularine	74.65 μM	48	3.5	0.001
PLC/PRF5	p16INK4a	zebularine	74.65 μM	24	2.7	0.001
PLC/PRF5	p16INK4a	zebularine	74.65 μM	48	3.7	0.001
PLC/PRF5	DNMT1	zebularine	74.65 μM	24	0.40	0.001
PLC/PRF5	DNMT1	zebularine	74.65 μM	48	0.18	0.001
PA-TU-8902	p14ARF	zebularine	98.82 μM	24	2.4	0.001
PA-TU-8902	p14ARF	zebularine	98.82 μM	48	2.7	0.001
PA-TU-8902	p15INK4b	zebularine	98.82 μM	24	2.3	0.001
PA-TU-8902	p15INK4b	zebularine	98.82 μM	48	2.5	0.001
PA-TU-8902	p16INK4a	zebularine	98.82 μM	24	2.1	0.001
PA-TU-8902	p16INK4a	zebularine	98.82 μM	48	2.4	0.001
PA-TU-8902	DNMT1	zebularine	98.82 μM	24	0.70	0.028
PA-TU-8902	DNMT1	zebularine	98.82 μM	48	0.45	0.001

## Conclusion

In conclusion, our finding demonstrated that zebularine inhibited PLC/PRF5 and PA-TU-8902 cell growth and induced apoptosis in these cell lines. The most likely mechanism underlying the zebularine inhibited cell growth and induced apoptosis involves down-regulation of DNMT1 and up-regulation of p16INK4a, p14ARF, and p15INK4b genes. This result suggests that zebularine may have wide therapeutic applications in hepatocellular carcinoma and pancreatic cancer.

## Author contribution

This study is a research work that reports the Effect of Zebularine on p16INK4a, p14ARF, p15INK4b, and DNA Methyltransferase 1 Gene Expression, Cell Growth Inhibition, and Apoptosis Induction in Human Hepatocellular Carcinoma PLC/PRF5 and Pancreatic Cancer PA-TU-8902 Cell Lines. It has not been previously submitted by any other journal, and all of the authors read and approved the final version of the manuscript. All authors generated the ideas and contributed to the writing of the manuscript, acquisition of data, analysis, and interpretation of data, and revised the article. All authors approved the final revision.
